# Implementation of quality medical physics training in a low‐middle income country — sharing experience from a tertiary care JCIA‐accredited university hospital

**DOI:** 10.1120/jacmp.v17i6.6553

**Published:** 2016-11-08

**Authors:** Ahmed Nadeem Abbasi, Wazir Muhammad, Amjad Hussain

**Affiliations:** ^1^ Department of Oncology, Aga Khan University Hospital (AKUH) Karachi Pakistan; ^2^ Pakistan Institute of Nuclear Science and Technology (PINSTECH) Islamabad Pakistan

To the Editor:

Our team wishes to share with the readers of a medical physics journal our experience of establishment of a two‐year radiation oncology physics postgraduate training program. Our purpose of sharing this experience is to encourage medical physics colleagues to come up with innovative ideas and to take up this challenge of improving the quality of medical physics training in order to improve the overall health‐care quality of professionals involved in the radiation oncology services of cancer patients in countries like Pakistan.

The Aga Khan University (AKUH), Pakistan's first private international university, is committed to the provision of education, research, and health care of international standard relevant to Pakistan and the region. In line with this vision, the University Hospital has established a state‐of‐the‐art Radiation Oncology facility. AKUH is the only hospital in Pakistan, and one of the few teaching hospitals in the world, to be awarded Joint Commission International (JCI) accreditation for achieving and maintaining highest international quality standards in health care. AKUH has also received the ISO 9001:2008 certification.

In the specialty of radiation oncology, medical physicists play an imperative role. They are skilled professionals with multidisciplinary responsibilities including, but not limited to, radiation safety, treatment planning, treatment delivery, and quality assurance in radiation therapy. A number of new modern radiotherapy units, such as Gamma Knife, Cyber Knife, Varian Trilogy, Elekta Synergy, have recently been installed in Pakistan and especially in Karachi. This makes the deficiency of skilled medical physicists even more critical in private sector. The AKUH has taken the initiative of ‘capacity building’ locally, with the help of international expertise, by establishing a two‐year Certificate Program in Medical Physics specializing in radiation therapy to meet the emerging needs of quality radiation therapy.

## PROGRAM OBJECTIVES

The objective of the program is to educate and to train students to a competency level sufficient to practice radiation oncology physics independently. This prepares the students for clinical practices in radiation therapy (RT) physics through a structured clinical trainings and didactic courses. The program is supervised and mentored by highly qualified clinical practitioners. Upon completion, the individual will be competent to assume clinical responsibility for medical physics‐related tasks in radiation therapy. Specifically, this enables students to have an appropriate familiarity, awareness, and understanding of the following issues:

• The role and responsibilities of the medical physicist within the multidisciplinary clinical team, with main focus on treatment planning, development, and implementation of quality assurance procedures and radiation safety.
• The processes of radiation therapy, availability and usage of state of the art technology, and development and implementation of novel clinical techniques in radiation therapy.
• Active participation in clinical research, teaching, and training.

## PROGRAM DESIGN AND CONTENTS

The program design is heavily influenced by the Canadian medical physics education system. The program objectives follow the guidelines of the American Association of Physicists in Medicine. The syllabus and the reference text are based on the IAEA recommendations.

The admission requirement for the Aga Khan University Hospital (AKUH) program is a Master's degree in physics with a minimal, if any, exposure to medical physics. This program was developed and implemented in 2009. The core contents of the program are regularly reviewed for revisions and improvements by the Education Committee of the Department of Oncology.

This structured program includes the following components:

**Didactic Courses:** Anatomy & physiology, basic physics of radiation therapy, radiation therapy methodology, clinical oncology, radiobiology, physics of medical imaging, advanced topics in radiation therapy physics, current trends in radiation therapy, seminar course, and journal club.

**Clinical Component:** Covers the competencies of the students in quality assurance, treatment planning, brachytherapy, radiation protection, and special techniques. Research & Development Projects: All the students participate in the departmental research and development projects, as well as those designed specifically for the certificate program. In addition, towards the final semester each student has to write a research thesis project as partial fulfillment of the certificate program requirements.

**Acquaintanceships:** This includes clinical rotations, as appropriate, in diagnostic radiology and nuclear medicine at AKUH. This may also include visits to radiotherapy centers outside AKUH to provide the trainees an overview of some of radiotherapy facilities not available inside AKUH: PET/CT, Cobalt‐60 teletherapy machine, SRS, IGRT and treatment planning systems other than Eclipse are a few examples. The aim is to broaden the understanding of the students towards medical physics. Another important objective of the rotation is to develop and improve communication skills required to establish working relationships with medical physicists and other health‐care professionals.

## EVALUATION AND CERTIFICATION

• Each trainee must maintain a full record of activities performed during the certificate program in terms of a portfolio, to be checked periodically by the mentor.
• Trainees must pass all the courses mentioned above.
• At the end of the first year, trainees will have to go through a peer review process (Peer Review A), covering the objectives to date.
• At the end of the final year, the students will need to complete another review process (Peer Review B), covering the entire certification requirements.
• The Peer Review involves a comprehensive oral exam taken by a panel of qualified medical physicists, oncologists, and radiation therapists. It may require the trainee to demonstrate hands‐on skills in different medical physics specialties. It also includes a written exam at the end of the final year.
• Successful completion of the two‐year program will lead to a Certificate in Medical Physics, attesting to the recipient's knowledge and technical skills required to carry out independently the functions of a medial physicist in radiation therapy.

## MENTORS AND FACILITATORS

There are six qualified medical physicists, a radiation safety officer, and four radiation oncologists available for teaching in this structured program.

## JOB OPPORTUNITIES

Karachi is the largest city in the country and is a home to several private sector hospitals that offer radiotherapy services. A large number of cancer patients is seen to in these public sector medical centers. Eighteen of these centers operate under the umbrella of the PAEC (Pakistan Atomic Energy Commission). Theses medical centers, however, hire medical physics graduates only from PIEAS (Pakistan Institute of Engineering and Applied sciences). In the private sector, several medical centers have been established with the provision of standard care services available to the cancer patients.

## COPYRIGHT

This work is licensed under a Creative Commons Attribution 3.0 Unported License.

